# Mitochondrial GWAS and association of nuclear – mitochondrial epistasis with BMI in T1DM patients

**DOI:** 10.1186/s12920-020-00752-7

**Published:** 2020-07-07

**Authors:** Agnieszka H. Ludwig-Słomczyńska, Michał T. Seweryn, Przemysław Kapusta, Ewelina Pitera, Samuel K. Handelman, Urszula Mantaj, Katarzyna Cyganek, Paweł Gutaj, Łucja Dobrucka, Ewa Wender-Ożegowska, Maciej T. Małecki, Paweł P. Wołkow

**Affiliations:** 1 Center for Medical Genomics OMICRON, Jagiellonian University Medical College, Kraków, Poland; 2 The Ohio State University Wexner Medical Center, Department of Cancer Biology and Genetics, Columbus, OH USA; 3 Department of Internal Medicine, Department of Computational Medicine and Bioinformatics, University of Michigan, Ann Arbor, MI USA; 4 Division of Reproduction, Poznań University of Medical Sciences, Poznań, Poland; 5 Department of Metabolic Diseases, University Hospital Kraków, Kraków, Poland; 6 Department of Metabolic Diseases, Jagiellonian University Medical College, Kraków, Poland

**Keywords:** Obesity, Mitochondria, Mitochondrial-nuclear interactions, GWAS

## Abstract

**Background:**

BMI is a strong indicator of complications from type I diabetes, especially under intensive treatment.

**Methods:**

We have genotyped 435 type 1 diabetics using Illumina Infinium Omni Express Exome-8 v1.4 arrays and performed mitoGWAS on BMI. We identified additive interactions between mitochondrial and nuclear variants in genes associated with mitochondrial functioning MitoCarta2.0 and confirmed and refined the results on external cohorts: the Framingham Heart Study (FHS) and GTEx data. Linear mixed model analysis was performed using the GENESIS package in R/Bioconductor.

**Results:**

We find a borderline significant association between the mitochondrial variant rs28357980, localized to MT-ND2, and BMI (β = − 0.69, *p* = 0.056). This BMI association was confirmed on 1889 patients from FHS cohort (β = − 0.312, *p* = 0.047). Next, we searched for additive interactions between mitochondrial and nuclear variants. MT-ND2 variants interacted with variants in the genes SIRT3, ATP5B, CYCS, TFB2M and POLRMT. TFB2M is a mitochondrial transcription factor and together with TFAM creates a transcription promoter complex for the mitochondrial polymerase POLRMT. We have found an interaction between rs3021088 in MT-ND2 and rs6701836 in TFB2M leading to BMI decrease (inter_pval = 0.0241), while interaction of rs3021088 in MT-ND2 and rs41542013 in POLRMT led to BMI increase (inter_pval = 0.0004). The influence of these interactions on BMI was confirmed in external cohorts.

**Conclusions:**

Here, we have shown that variants in the mitochondrial genome as well as additive interactions between mitochondrial and nuclear SNPs influence BMI in T1DM and general cohorts.

## Background

Mitochondria are organelles whose main role is energy production. They are the only organelles that contain their own genome. The mitochondrial genome is a double stranded 16.5 kb long molecule which resembles that of an alpha-proteobacterium [1]. The two strands differ in nucleotide (G + T) composition – the guanosine-rich strand is named the heavy (H) strand; the other, cystosine-rich strand is called the L-strand (light strand). Like a free-living eubacterium, mitochondrial genome contains no introns and minimal intergenic regions, although it contains an approximately 1000 nucleotide non-coding control region (displacement loop or D-loop) where the origin of replication of the H-strand as well as promoters of transcription, both for H- and L-strands are localized. mtDNA codes for 37 genes. Among them 13 code for polypeptides, while the remainder - 2 rRNAs (12S and 16S) and 22 tRNAs – are necessary for mitochondrial protein synthesis. All 13 mRNAs code for subunits of oxidative phosphorylation (OXPHOS) complexes. The rest of the peptides needed to build the electron transport chain (ETC), as well as to maintain mitochondrial functioning, are nuclear-encoded [2]. It has been shown that mitochondrial function correlates with cells’ metabolic state and can influence obesity [3].

Obesity is a severe epidemic world-wide [4]. Current trends suggest that by the year 2030 more than 50% of Americans will be obese [5], while morbid obesity will affect even 10% of the UK population [6]. Type 1 diabetics have seen an even greater increase in obesity incidence than is observed in the general population, [7], with obesity in type 1 diabetics becoming two times more prevalent over the last 30 years [8, 9]. Obesity in type 1 diabetics has been associated with severe complications, especially in patients undergoing intensive therapy [10]. Such a burst in obesity prevalence can, in part, be attributed to lifestyle and to higher doses of insulin [11], however obesity is also highly heritable. Twin studies suggest that 40–70% of variability of BMI, the most popular measure to assess obesity, can be attributed to genetic variation [12]. Even though large scale genome wide association studies (GWAS) including hundreds of thousands of individuals and millions of autosomal single nucleotide variants have been performed, they led to discovery of only around 100 genetic variants associated with BMI [13–15]. Thus, a substantial part of genetic variation that influences BMI remains to be discovered. As with most complex traits, most of the associated variants are non-coding (making explanation of their role in obesity even more difficult) [16].

Ultimately, obesity results from an imbalance between energy intake and its expenditure. Since interaction and communication between nuclear and mitochondrial genomes is indispensable for normal cell function [17, 18], it seems reasonable to look for interactions between SNPs in the nuclear genome and SNPs in the mitochondrial genome associated with obesity [19–21]. Here, we have performed mitochondrial GWAS as well as a study of genetic interactions between mitochondrial and nuclear variants which are localized to genes known to have an influence on mitochondrial functioning, associating variants in both genomes with BMI.

## Methods

### Patients

Patients were recruited either in Department of Metabolic Diseases University Hospital in Krakow or in Division of Reproduction Department of Obstetrics, Gynecology and Gynecological Oncology, Poznan University of Medical Sciences. All patients enrolled to the study were young women with type 1 diabetes (T1D) and on insulin treatment, who were pregnant or were trying to conceive. Whole blood samples were drawn and stored at − 80 °C. This study was approved by the Bioethical Committees of the Jagiellonian University and Poznan University of Medical Sciences and performed according to the Helsinki Declaration. Written informed consent was collected from all patients.

### Genotyping

DNA was extracted from whole blood with the use of automated nucleic acid extraction system Maxwell (Promega). Five hundred twenty-seven samples were genotyped on Illumina Infinium Omni Express Exome-8 v1.4 arrays according to manufacturer’s instructions.

### Quality control (QC)

Genotypes were called by GenomeStudio software Genotyping module (version 2.0, Illumina Inc.) according to manufacturers’ instructions (Technical Note: Genotyping, Infinium® Genotyping Data Analysis, www.illumina.com). Briefly, we removed all samples with 10%GenCall score < 0.4, Call Rate < 0.95 and discordant sex information. Then, all SNPs on chrX, chrM, chr0 were removed and hard cut off metrics was applied. Next, we manually curated 201 variants on chrM also in Genome Studio, recalculated statistics and excluded samples with Call Rate < 0.99 (180 remained in the analysis). Remaining nuclear genotypes for 940,911 SNPs and 513 samples were exported as custom report using plink (version 1.09) [22].

Second step of quality check was performed using custom rscript in Rstudio (version 1.1.383) [23] with R (version 3.4.2) [24] according to plink manufacturers’ instructions and KING [25]. Briefly, we removed duplicates with lower Call Rate and samples with cryptic kinship, equivalent to third-degree relatives and higher (kinship coefficient > 0.0442). In addition, we also checked for heterozygosity rate of autosomal SNPs and removed variants with deviation from the Hardy-Weinberg equilibrium (HWE) with *P* < 1e10–5. The last part of QC was based on EIGENSOFT [26, 27] in order to determine population structure in our samples based on HapMap3 dataset [28]. Samples that failed to qualify as European population (CEU) were removed. Finally, 476 individuals with 940,374 nuclear variants passed QC and were included in the analysis, however, due to lack of metadata only 435 patients were included in the final analysis. Apart from nuclear variants, 180 mitochondrial variants were included in the analysis.

### Imputation

The QC-filtered genotype data were checked against reference panel of the Haplotype Reference Consortium (HRC r1.1 2016, ftp://ngs.sanger.ac.uk/production/hrc/HRC.r1-1/HRC.r1-1.GRCh37.wgs.mac5.sites.tab.gz*)* with [29] “HRC/1KG Imputation Preparation and Checking Tool” (v.4.2.9, https://www.well.ox.ac.uk/~wrayner/tools) to exclude strand coding issues during the imputation step. Imputation of QC-filtered genotypes was performed on Michigan Imputation Server (using Minimac3) [30] with the HRC r1.1 reference panel. Phasing was performed with ShapeIT (version 2.r790) [31]. The Minimac3 output dosage files were then converted to hard calls in PLINK. For further analysis imputed MitoExome variants were chosen based on MitoCarta2.0 gene localization [32]. The phenotypic data were available for 435 patients which were included into the final analysis.

### Framingham heart study cohort

The genotype and clinical phenotype files were downloaded from dbGaP (project #5358; accession number phs000007). Only the genotypes corresponding to the SHARe substudy were used (obtained via OMNI5M genotyping array, phg000256). In the GLMs, the following covariates were used: age (phv00177930), gender (phv00177929) and blood glucose level (phv00007558). Our access was approved by the Ohio State University IRB (Protocol #2013H0096) (see Availability of data and materials).

### GTEx

Tissue specific RNA sequencing data was acquired from the Genotype and Tissue Expression Project (GTEx -v7) via the website gtexportal.org. The phenotye data was accessed via dbGaP Project #5358 (dbGaP accession number phs000424).

RNA sequencing had been generated using poly-adenylated priming with reads aligned to HG19, Gencodev12. For further details see Lonsdale et al. [33] and the GTEx website (http://www.gtexportal.org/home/documentationPage). We considered subcutaneous adipose and thyroid gland. Testing for eQTLs was performed via the GTEx online search tool [34]. The GLM approach was utilized to test the association between BMI (phv00169070) (adjusted for age (phv00169063) and sex (phv00169062)) and expression of selected mitochondrial and/or nuclear mRNAs (see Availability of data and materials).

### Generalized linear model (GLM) analysis

The linear mixed model analysis was performed using the GENESIS package in R/Bioconductor [35]. In brief, first the genetic relatedness matrix was estimated via the PC-AiR and PC-Relate methods, subsequently a linear model was built and Wald’s test was used to assign significance. The interactions between variants was modeled via the option ‘ivars’ in function ‘assocTestMM’. All *p*-values reported are non-adjusted for multiple testing.

### Filtering variants likely to be involved in epistatic interactions

For the analysis of epistatic interactions between the mitochondrial and nuclear (whole genome) variants a filtering step was performed to increase the likelihood of detection and limit the number of hypotheses tested. The details of our approach are presented in the Supplementary Methods. Our concept is closely related to the notion of variance QTLs [36, 37] i.e. variants which are associated with the variability of a trait. In brief, we compare the relative dispersion (conditional on the given nuclear SNP) of the residuals in two GLMs: (1) BMI ~ covariates+mito_variant, and (2) BMI ~ covariates+mito_variant+nuc_variant. The basic assumption is that if the residuals of the second GLM have higher relative (conditional on the nuclear SNP) dispersion that the residuals from the first model, then the nuclear SNP is more likely to interact with a variable (not necessarily the mitochondrial genotype) in the context of BMI. We test this assumption (together with our filtering approach) by means of simulation and random subsampling (from real-life data) and will present complete results in a forthcoming paper (results, data and code available upon request). Below we show that this approach allows to select variants likely to interact epistaticaly with the MT-ND2 rs28357980 variant (Table 5). At the same time, a more extensive analysis based on the FHS genotype data proves that our method allows to select subsets of variants which are enriched in epistatic interactions in the context of BMI.

## Results

### Single SNP analysis – mitoGWAS

The cohort consisted of 435 young women with mean age of 28.5 years. Most of them were of normal weight and have never been pregnant before being included to the study. Mean disease duration was 12 years, while median insulin daily dose equaled 40 IU. Anthropometric data are presented in Table 1.

**Table 1:** Antropometric data of T1DM cohort

	Min	1st Q	Median	Mean	3rd Q	Max
Age (years)	17	25	28	28.51	32	48
Disease duration (years)	0.50	5	12	12.07	18	36
Daily insulin dose (IU)	1	30	40	41.62	52	120
# of pregnancies	1	1	1	1.44	2	4
Before pregnancy BMI [kg/m^2^]	16.20	21.05	23.38	23.97	25.95	45.17

Forty-two mitochondrial variants were included in the analysis after filtering according to alternative allele frequency (Table S1a). Borderline significant nominal association with lower BMI was shown for 4 mitochondrial variants. Three of them were localized to non-coding part of mitochondrial genome – one to mitochondrial ribosomal gene MT-RNR2 and two to MT-tRNAs (Arg, Thr). The last one – rs28357980 was localized to MT-ND2 gene (MitoA4917G), which is part of the complex I of electron transport chain. The variant leads to amino acid change from Asparagine to Aspartic acid in position 150 of MT-ND2 protein. Results of mitoGWAS on BMI in T1DM cohort are presented in Table 2.

**Table 2:** mitoGWAS results on BMI in T1DM cohort

Probe_ID	rs_number	MAF	Mitochondrial localization	Gene	βSNP	SE	*p*-value
exm-rs28358279-132_T_R_1990486714	rs28358279	10.8%	10,463	MT-TR	−0.73	0.355	0.040
**exm2263338-0_B_R_1978044975**	**rs28357980**	**10.3%**	**4917**	**MT-ND2**	**−0.69**	**0.362**	**0.056**
exm2263337-0_B_R_1978044973	rs527236198	9.9%	15,928	MT-TT	−0.70	0.370	0.056
exm2216232-0_T_F_1955482039	rs2897260	9.6%	1888	MT-RNR2	−0.70	0.374	0.058

An analogous analysis of 87 mitochondrial variants (Table S1b) on 1889 subjects gathered in Framingham Heart Study (validation cohort) was performed. For the analysis, only the first measurement of BMI was used. The cohort consisted of 1037 women and 852 men. Their mean age was 34.6, half of them were normoglycemic. Median BMI for this cohort was 24.28 (Table 3).

**Table 3:** Antropometric data of FHS cohort

	Min	1st Q	Median	Mean	3rd Q	Max
Age (years)	5	28	34	34.6	41	59
Blood glucose levels (mg/dL)	65	95	100	99.98	105	214
BMI [kg/m^2^]	13.52	21.80	24.28	24.77	27.12	50.98

Nominal associations with BMI were found for 7 variants. All nominally associated variants led to BMI decrease. Four of them were coding, the rest was localized to rRNA and tRNAs.

Among coding variants, we have found the same variant rs28357980 localized to MT-ND2 gene for which an association with BMI in T1DM cohort was found. Results of mitoGWAS on BMI in FHS cohort are presented in Table 4.

**Table 4:** mitoGWAS results on BMI on FHS cohort

Probe_ID	rs_number	MAF	Mitochondrial localization	Gene	βSNP	SE	*p*-value
MitoG3012A	rs3928306	45.8%	3010	MT-RNR2	−0.241	0.113	0.033
**MitoA4918G**	**rs28357980**	**19.1%**	**4917**	**MT-ND2**	**−0.312**	**0.157**	**0.047**
MitoT9900C	rs41345446	3.8%	9899	MT-CO3	−0.785	0.338	0.020
MitoA11252G	rs869096886	35.8%	11,251	MT-ND4	−0.241	0.123	0.051
200,610–74	rs193302994	36.1%	15,452	MT-CYB	−0.242	0.123	0.049
MitoG15929A	rs527236198	18.2%	15,928	MT-TT	−0.396	0.163	0.015
MitoA16164G	rs41479950	4.6%	16,163	MT-TP	−0.689	0.310	0.027

### MT-ND2 gene - nuclear genes interactions

Each of the analysis we performed (mitoGWAS in both cohorts) directed us towards MT-ND2 gene. Since there must be a tight communication between the mitochondrial and the nuclear genome in order to achieve effective energy management [38–41], we looked for the interactions (i.e. epistasis between genetic variants in GLMs) between the two genomes. The results are presented in Table 5.

**Table 5:** List of MT-ND2 interactions with nuclear variants in T1DM cohort

Probe_ID	rs_number	MitoMAF	Nuclear SNP ID	Nuclear MAF	Nuclear localization	*p*-value
exm2263338-0_B_R_1978044975	rs28357980	10%	rs17380506	7.0%	BRINP2	1.04e-07
			rs10166792	8.0%	HS1BP3	4.00e-06
			rs17170333	6.9%	Intergenic, nearest BMPER	2.45e-06
			rs4739037	6.9%	NKAIN3	1.04e-07
			rs12541993	6.9%	NKAIN3	1.04e-07
			rs930840	6.2%	LOC107986946	2.20e-06
			rs4737627	6.0%	Intergenic, nearest NKAIN3	8.45e-07
			rs7020872	5.4%	Intergenic, nearest SLC25A6P2	2.79e-07
			rs10758117	17.3%	Intergenic, nearest SLC25A6P2	2.51e-06
			rs10970789	17.3%	Intergenic, nearest SLC25A6P2	2.61e-06
			rs10970792	21.1%	Intergenic, nearest SLC25A6P2	3.25e-06
			rs10970828	17.4%	Intergenic, nearest SLC25A6P2	2.55e-06

We also performed such an analysis on FHS cohort and found 53 nuclear variants which interact with rs28357980 (MT-ND2), several of which served as eQTLs. When these genes were subjected to GoTerm analysis we found an enrichment of terms associated with mitochondria (Mitochondrion padj = 9.56e-05, Mitochondrial part padj = 2.62e-04, Mitochondrial matrix padj = 2.52e-03).

### MT-ND2 gene interactions within MitoCarta

The above results (i.e. the enrichment of genes involved in the functioning of mitochondria among the ones which have variants involved in epistatic interaction with rs28357980) guided us to further explore the potential for epistasis between the MT-ND2 variant and nuclear SNPs located in the MitoCarta genes, which are known to be associated with mitochondrial pathways (Table S2a, b). Most significant interactions for MT-ND2 were associated with TCA cycle and respiratory electron transport, metabolism of amino acids and mitochondrial biogenesis (Table S3). The last category can potentially have the most crucial influence on mitochondrial functioning and can have profound significance for energy metabolism. Reactome lists 17 genes (POLRMT, TFB1M, TFAM, MTERF, PERM1, TFB2M, ATP5B, SSBP1, SIRT3, POLG2, PPARGC1A, CYCS, NRF1, ALAS1, PEO1, GABPA, ESRRA) which constitute this pathway, five of which are present in the results of our analysis. The list of MT-ND2 variants interactions within this pathway is presented in Table 6.

**Table 6:** List of MT-ND2 interactions within mitochondrial biogenesis pathway

Probe_ID	rs_number	MitoMAF	Nuclear SNP ID	Nuclear MAF	Nuclear localization	*p*-value
exm2263338-0_B_R_1978044975	rs28357980	10.0%	rs113919457	19.3%	SIRT3	0.0016
			rs11607019	19.3%	SIRT3	0.0016
exm-rs3021088-132_B_R_1990477615	rs3021088	5.1%	rs2950393	28.4%	ATP5B	0.0028
			rs10215217	28.9%	CYCS	0.001
			rs41542013	15.0%	POLRMT	0.0004
			rs6701836	37.0%	TFB2M	0.024

We looked closer into these interactions and found that variant rs3021088 of MT-ND2 gene interacted with variants in TFB2M (rs6701836) and POLMRT (rs41542013) genes, both of which are necessary for mitochondrial transcription. TFB2M is a mitochondrial transcription factor which, together with TFAM, creates transcription promoter complex and enables transcription by mitochondrial polymerase POLRMT (Figure S1).

When looking at the interaction between TFB2M nuclear variant – rs6701836 and MT-ND2 mitochondrial variant – rs3021088 we found that the combination of the two led to BMI decrease [(nuc_eff(single model) = − 0.0660, nuc_pval(single) = 0.857, joint_eff(inter) = 0.0874, nuc_pval(inter) = 0.0773, inter_eff = − 2.225, inter_pval = 0.0241]. Neither nuclear nor mitochondrial variant on its own had such an effect [p(nuc) – *p* = 0.8577, p(mito) – *p* = 0.116].

Interaction between POLRMT variant rs41542013 and MT-ND2 mitochondrial variant rs3021088 led to BMI increase [nuc_eff(single model) = 0.296, nuc_pval(single) = 0.546, nuc_eff(inter) = − 0.085, joint_pval(inter) = 0.0016, inter_eff = 4.015, inter_pval = 0.0004]. None of these variants on its own was not associated with BMI.

Thus, we have found an example of negative interaction between MT-ND2 and TFB2M and positive interaction between MT-ND2 and POLRMT variants.

### Functional significance

Next, we assessed the functional significance of these interactions. Mitochondrial rs3021088 is a missense variant which leads to Alanine to Threonine substitution in position 331 of MT-ND2 protein.

**Fig. 1:**
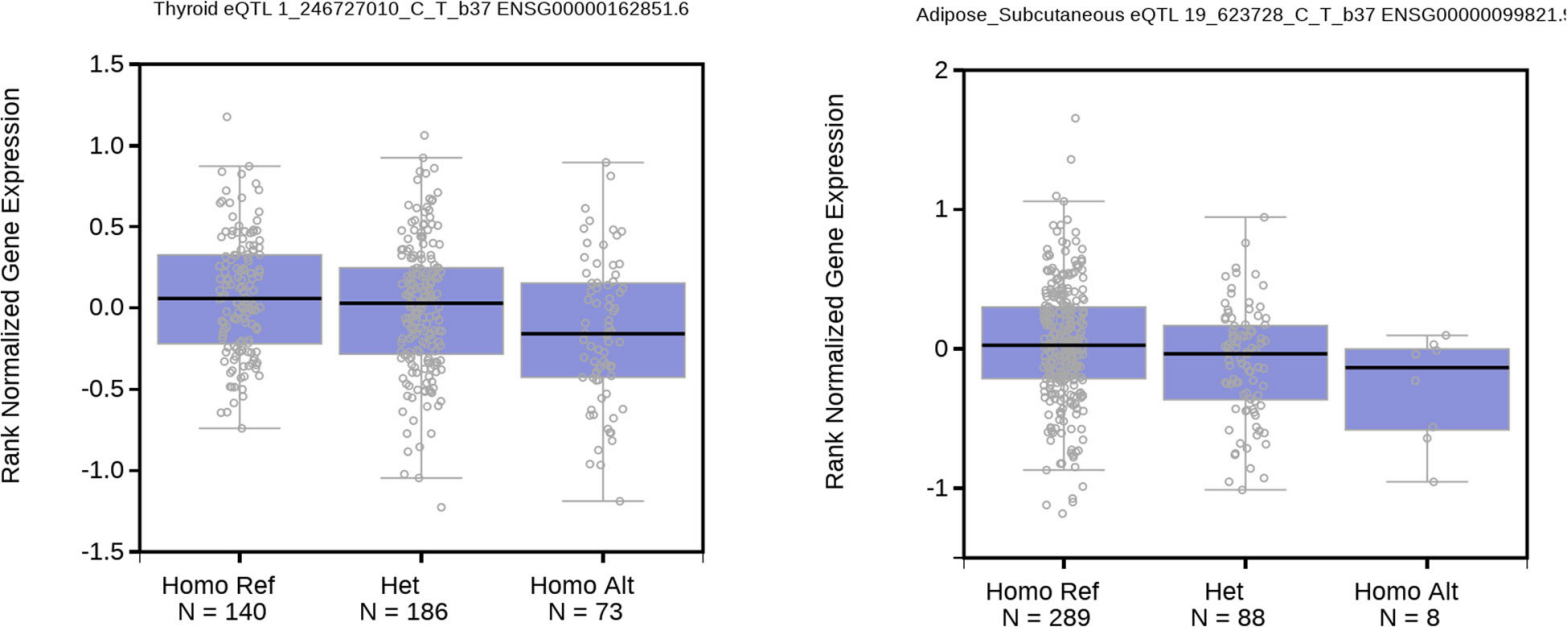
Downregulating effect of rs6701836 on expression of TFB2M gene in thyroid and rs41542013 on POLRMT expression in subcutaneous adipose tissue

eQTL data (GTEx) for nuclear rs6701836 showed that it influenced TFB2M expression in thyroid gland. POLRMT variant rs41542013 in subcutaneous adipose tissue leads to lower POLRMT expression (Fig. 1). In GTEx data, mRNA levels of MT-ND2 and TFB2M correlated with higher BMI [p(nuc) = 0.0269, eff_nuc = 2.465e-01, p(mito) = 0.0244, eff_mito = 2.243e-04] in liver tissue, however, their interaction led to decrease of BMI [*p* = 0.0308, inter_eff = − 1.009e-05]. In GTEx data, mRNA levels of MT-ND2 and POLRMT on its own correlated with lower BMI [p(nuc) = 0.0492, eff_nuc = − 2.386e-01, p(mito) = 0.0688, eff_mito = − 2.233e-04] in subcutaneous adipose tissue, however, their interaction led to increase of BMI [*p* = 0.0235, inter_eff = 1.023e-05]. Taken together, the interactions on the mRNA level are in line with what was discovered in the T1DM cohort.

### Validation cohort

We also used FHS as a validation cohort to confirm the epistatic interactions between SIRT3, ATP5B, CYCS, TFB2M or POLRMT and MT-ND2.

We were able to confirm the interactions and their influence on BMI for SIRT3 and ATP5B genes. For SIRT3 we have found an interaction between mitochondrial variant localized to 5460 position (MAF 10.1%) and two nuclear variants – rs11602954 (*p* = 0.03) and rs11606393 (*p* = 0.028). These variants are in LD with each other and with previously described variants in SIRT3 (in Table 6). For ATP5B gene we have found that mitochondrial variant localized to position 4769 interacted with seven variants (rs2255074, rs1107479, rs7973157, rs2950390, rs2958154, rs2290893, rs2035081) which were in LD with rs2950393.

We looked closer into interactions of MT-ND2 and TFB2M or POLRMT. For TFB2M gene, we have found two interactions. Variant rs10924779 interacted with MT-ND2 variant localized to 5460 position (MAF = 10.1%). Their interaction leads to BMI increase (*p* = 0.037), while each of these variants on their own did not have an influence on BMI. Variant rs4654291 interacts with the same mitochondrial variant and leads to BMI increase (*p* = 0.045), while each of these on its own does not affect BMI. For POLRMT gene we have identified an intronic variant rs10411491 (MAF = 3%) which interacted with mitochondrial variant positioned at 4647 (MAF = 4.8%). Their interaction led to BMI increase, although due to low intronic variant MAF it did not reach statistical significance.

Thus, we can conclude that interactions between TFB2M or POLRMT and MT-ND2 gene might affect BMI.

### Whole genome MT-ND2 interactions

Next, we investigated whole genome MT-ND2 interactions. All variants which entered statistically significant interactions with MT-ND2 gene in FHS cohort and T1DM cohort were listed (Table S4a, b). These variants were compared to results gathered in GWAS catalog. Among 366 nuclear variants which interacted with mitochondrial variant rs28357980, 5 were found in GWAS Catalog and 2 of them met genome wide significance threshold. Out of 1157 variants which interacted with mitochondrial variant rs3021088, 35 were listed in GWAS Catalog and 13 were significant at the level of p < e10–09 (Table S4c). According to GWAS Catalog these variants were associated, among others, with blood metabolite levels, blood protein levels, LDL and total cholesterol, bone mineral density, age-related hearing impairment.

## Discussion

Functioning of mitochondria have a profound effect on whole organism. The variants within mitochondrial as well as nuclear genomes might have an influence on the levels of ATP produced within mitochondria. ATP depletion can induce endoplasmic reticulum (ER) stress and lead to reactive oxygen species (ROS) generation, which later on will result in mitochondrial DNA damage and create a vicious cycle of mitochondrial inefficacy. Here we have looked for mitochondrial variants and their interaction with nuclear variants which may be associated with BMI in the general population as well as in T1DM patients.

Our mitoGWAS analyses have shown that both protein coding and non-coding variants are associated with BMI. We have however looked closer into the former, as they might directly influence electron transport chain (ETC) and cells energy production. The analysis of T1DM cohort led to discovery of a variant in MT-ND2 gene. The predictive algorithms do not suggest that rs28357980 (G4917A) might be damaging (SIFT - 0.09, PolyPhen – 0.06), however the variant has been shown to be associated with multiple sclerosis [42] and colorectal cancer [43]. The G4917A variant is one of the defining variants of haplogroup T, which in 2013 was shown to be a risk factor for morbid obesity [44]. Moreover, the variant is non-synonymous and affects the protein by substitution of highly conserved Aspartate at amino acid 150 to an Asparagine. What is more interesting, our analysis of the validation cohort (FHS) confirmed the association of the same variant with BMI.

rs28357980 (G4917A) is localized to MT-ND2 gene which is part of complex I of ETC. It is the first site of oxidative phosphorylation. Complex I is built of 46 proteins, 7 of which are mitochondrially coded (ND1, ND2, ND3, ND4, ND4L, ND5, ND6) and form very hydrophobic subunits within mitochondrial membrane. Disruptive mutations in ND subunits are commonly found as somatic mutations in tumors, but are not found as germline mutations associated with human diseases, due to their lethality [45]. Previous studies have already associated variants in these genes with obesity. A paper from 2014 has shown, apart from associations with two other positions, an association between three variants in complex I genes – MT-ND1, MT-ND2 and MT-ND4L [46]. Moreover, the variant C5178A in MT-ND2 gene was shown to lead to lower incidence of autoimmune diabetes. The A allele was protective against both autoimmune and alloxan-induced free radical–mediated diabetes in mice, possibly by suppressing ROS production at the β-cell level [47–49]. Apart from metabolic disorders, mutations in genes of complex I of ETC were shown to be associated with childhood acute lymphoblastic leukemia or were shown to be a poor prognostic factor in oral cancer [50].

Since several GWAS data show that a substantial part of genetic variability of obesity is still unknown one might suspect that it is hidden in more complex associations, meaning genetic interactions. It is known that mitochondrial functioning is a result of anterograde and retrograde signaling between mitochondrial and nuclear genomes. Most of the studies performed by now, did not analyze point mitochondrial variants, but conplastic animals in which nucleus from one organism was fused with cytoplasts of the other. Such experiments have shown that introduction of exogenous mitochondria (in which mitochondrial genome differed from the original by one or few nucleotides) influenced organismal phenotype. For example, conplastic rats where shown to have impaired glucose tolerance, while mice were more resistant to experimental autoimmune encephalomyelitis [51], had disrupted activity of the components of TCA cycle [52] or altered mitochondrial and cellular adaptation during aging [53]. Moreover, the mutation in MT-ND2 gene (C4738A) in mouse fibroblasts led to significantly higher mitochondrial complex I activity, enhanced ATP production, reduced ROS production with similar MT-ND2 protein expression levels [54]. A lot of studies have shown that even a point mutation in mitochondrial genome which was introduced onto another nuclear background led to severe mitochondrial dysfunction. A point mutation in ATP8 gene (7778 G/T) in C57BL/6 N-mt^FVB/N^ mice led to lower insulin secretion in isolated islets after glucose stimulation when compared with C57BL/6 N-mt^AKR/J^ mice. It also had reduced mitochondrial function in brain, spleen and liver [55] as well as showed a 3-fold higher generation of mitochondrial ROS production compared to C57BL/6 N-mt^AKR/J^ mice [56].

However, since mitochondrial genome mutates faster than nuclear (for example due to ROS proximity) the incompatibility between the two genomes might occur during organismal lifetime. What is more, these interactions can also be influenced by the environment.

Thus, we have looked into epistatic interactions of MT-ND2 variants. We therefore checked the potential for interactions of MT-ND2 variant located in position 4917 in T1DM cohort. Our whole genome analysis has shown that it possibly interacts with 12 variants, most of which might influence energy metabolism or processes that have been shown to be associated with obesity. One variant was located in HS1BP3 gene. HS1BP3 is known to be localized to mitochondria, be involved in autophagy by inhibiting phospholipase D and its overexpression leads to apoptosis [57–59]. We also found four variants, in LD with each other, located either within or next to NKAIN3 gene, which turned out to be eQTLs for GGH gene. Gamma glutamyl-hydrolase is an enzyme involved in folate metabolism, while lower folates levels were associated with reduced insulin sensitivity and obesity [60–62]. Moreover, we identified 5 intergenic variants located near SLC25A6P2 gene, which is a mitochondrial transporter family [63–65]. The analysis of rs28357980 (MT-ND2) interactions in FHS also pointed us toward analysis of nuclear mitochondrial genes.

The Reactome analysis done on MitoCarta genes has shown an enrichment of interactions that are associated with mitochondrial biogenesis. Our data show that POLRMT and TFB2M variants interact with variants in MT-ND2 and affect BMI. POLRMT and TFB2M are genes that act in concert to perform mitochondrial transcription and replication thus variation within their sequence, and simultaneously in the mitochondrial DNA sequence, can have an influence on mitochondrial and gene copy number as well as influence efficacy of the two processes [66–68]. Since it is very difficult to assess the significance of such interactions we also looked into all interactions of MT-ND2 variants and checked whether nuclear variants which interacted with mitochondrial MT-ND2 gene were listed as significant in any GWAS study performed until today, as we believed this would strengthen our findings. Our analysis has confirmed that some of the nuclear variants were significantly associated with traits which are part of obesity phenotype, e.g. cholesterol level, blood metabolites level or with diseases which are known to be influenced by mitochondrial deficiencies e.g. hearing loss.

## Conclusions

In conclusion, here we find that rs28357980 localized to MT-ND2 gene of mitochondrial genome is associated with BMI both in T1DM and in general cohort. What is more, we show that genetic epistasis might influence obesity phenotype by interaction of variants in MT-ND2 gene with nuclear variants in genes responsible for mitochondrial replication and transcription.

## Supplementary information

**Additional file 1.** Supplementary Methods.

**Additional file 2: Table S1a.** List of mitochondrial variants included in the analysis in T1DM cohort. **b**. List of mitochondrial variants included in the analysis in FHS cohort.

**Additional file 3: Table S2a.** All interactions of MT-ND2 gene with MitoCarta genes in T1DM cohort. **b**. Interactions of MT-ND2 gene significant after all corrections of MT-ND2 variant rs28357980 in FHS (MAF > 0.05, FDR < 0.05).

**Additional file 4: Table S3.** List of most significant associations of MT-ND2 gene.

**Additional file 5: Table S4a.** All interactions of MT-ND2 gene genome wide in T1DM cohort. **b**. All interactions of MT-ND2 gene genome wide in FHS cohort. **c**. Significant associations of MT-ND2 variants in GWAS Catalog.

**Additional file 6: Figure S1.** MitoGWAS on BMI type 1 diabetes patients and additive interactions between mitochondrial and nuclear variants in T1DM patients and FHS cohort.

## Data Availability

Sequence data has been deposited at the European Genome-phenome Archive (EGA), which is hosted by the EBI and the CRG, under accession number EGAS00001004408. Further information about EGA can be found on https://ega-archive.org “The European Genome-phenome Archive of human data consented for biomedical research” *(*http://www.nature.com/ng/journal/v47/n7/full/ng.3312.html*).* The FHS datasets were accessed via dbGaP (project #5358; accession number phs000007). The phg000256 OMNI5M SHARe dataset (access pends appropriate approval) was used for analysis. To access phg000256 dataset please use the following link in the dbGAP advanced search: (https://www.ncbi.nlm.nih.gov/gap/advanced_search/?OBJ=genotype&TERM=phg000256&COND=%7B%22study_name_accession_combo%22:%5B%22NHLBI%20Framingham%20SNP%20Health%20Association%20Resource%20(SHARe)%20%20(phs000342.v19.p12)225D%7D), which allows to look for OBJ = genotype TERM = phg000256 in the study phs000342.v19.p12. The following variables were used for analysis: age (phv00177930) and sex (phv00177929) - both belonging to pht003099, as well as BMI (calculated from height: phv00007570 and weight: phv00007569 - belonging to pht000030). Tissue specific RNA sequencing data was acquired from the Genotype and Tissue Expression Project (GTEx -v7) via the website gtexportal.org. The phenotye data was accessed via dbGaP Project #5358 (dbGaP accession number phs000424). The variables used for glm analysis using the GTEx project data were: BMI (phv00169070), age (phv00169063) and sex (phv00169062), all in pht002742. The QC-filtered genotype data were checked against reference panel of the Haplotype Reference Consortium (HRC r1.1 2016, ftp://ngs.sanger.ac.uk/production/hrc/HRC.r1-1/HRC.r1-1.GRCh37.wgs.mac5.sites.tab.*gz*)* with “HRC/1KG Imputation Preparation and Checking Tool” (v.4.2.9,*https://www.well.ox.ac.uk/~wrayner/tools) to exclude strand coding issues during the imputation step. Imputation of QC-filtered genotypes was performed on Michigan Imputation Server (using Minimac3) with the HRC r1.1 reference panel. All data used in this study are deposited in public repositories. The data are publicly available and access requires bioethics committee approval. The researchers who want to gain access to these data are encouraged to apply to the respective Institutional Review Boards. The sensitive data, including those which allow identification of individuals are available to anyone upon approval based solely on bioethics ground.

## Abbreviations

BMI: Body mass index
T1DM: Type 1 diabetes
SNP: Single nucleotide polymorphism
mtDNA: mitochondrial DNA
ETC: Electron transport chain
GWAS: Genome wide association study
MAF: Minor allele frequency
GLM: Generalized linear model
eQTL: Expression quantitative trait loci
TCA: Tricarboxylic acid cycle
