# Supplementary material for: Mitochondrial GWAS and association of nuclear – mitochondrial epistasis with BMI in T1DM patients
Source: BMC Med Genomics. 2020 Jul 7;13:97. doi: 10.1186/s12920-020-00752-7 (PMC7341625; doi:10.1186/s12920-020-00752-7)
Supplement: Supplementary file 1 — Additional file 1. Supplementary Methods. [file 12920_2020_752_MOESM1_ESM.docx]

Supplementary Methods.

1. **Variability QTL detection.**

The basic assumption for this analysis is that variants likely to be involved in non-linear interactions (including epistasis) are associated with increased variability of the trait. For the identification of variants which increase the variability of a trait, we take a non-parametric approach. Therefore, we do not rely on the notion of heteroskedasticity in GLMs, as the authors of PMC3914615, in order to make our results less affected by the departure from normality of the dependent variable. At the same time, we wish to make the least number of assumptions on the distribution of the dependent variable, thus we do not make use of the low-dimensional (parametric; for example second-order) representation to model the trait (for reference on such methods see for example PMC6288835).

We aim to detect variants which increase the variability of the trait in the context of the main effects by means of comparing the conditional entropies of the residuals in GLMs.

Assume that a trait (Y_i, i=1,...,m) and a set of independent variables (X_{ij}, i=1,...,n; j=1,2,...,n) follows some distribution and two genetic predictors (binary variables for simplicity) are given (Z_{i,j}, i=1,...,m; j=1,2). Consider two GLMs:

(1) Y_i ~ X_{i,1} + ... + X_{i,n} + Z_{i,1},

(2) Y_i ~ X_{i,1} + ... + X_{i,n} + Z_{i,1} + Z_{i,2},

and denote by (R_{i,j}, i=1,...,m; j=1,2) the residulas form the first and the second model respectively. The main idea of this approach is to study the conditional entropy of the residuals of these two models (under the condition of Z_{i,2}=1). In other words, by the chain rule for Shannon entropy, the total entropy may be decomposed into two distinc parts – the entropy conditional on Z_{i,2}=0 and on Z_{i,2}=1. If the second genetic predictor increases the variability of the trait in any of the GLMs, then the latter conditional entropy is larger then the former, i.e.

S(R_{i,j}|Z_{i,2}=0) < S(R_{i,j}|Z_{i,2}=1), for j=1 or j=2.

At the same time, the (possible) dependence of the residulas (R_{i,1}, i=1,...,m) on the values of (Z_{i,2}, i=1,...,m) may introduce a bias in the evaluation of the impact of the genetic prodictor on the variability of the trait. Therefore, we additionally compare the ratios of the conditional entropies form the two GLMs – i.e. we cosider the statistics

T(Y,X,Z) = (S(R_{i,2}|Z_{i,2}=1)/S(R_{i,2}|Z_{i,2}=1)) - (S(R_{i,1}|Z_{i,2}=0)/S(R_{i,1}|Z_{i,2}=1)).

Intuitively, whenever T>0, then the variability of the residuals in the second model is more affected by the value of the second genetic predictor, then the variability of the residuals from the first model. The null distribution of this statistics may be estimated by means of permutation testing for large cohorts.

For the identification of nuclear variants likely to be involved in interactions in the context of BMI, we discretize the residuals according to equal-frequency binning and take the intersection of two sets of variants for testing for epistasis: (a) all nuclear variants with T>0 in the T1DM cohort, and (b) all nuclear variants with T>0 in the FHS Offspring cohort. This approach allows us to limit the number of hypotheses tested to k=141648 variants with MAF>0.05.
