# Supplementary figures and images for: Mitochondrial GWAS and association of nuclear – mitochondrial epistasis with BMI in T1DM patients
Source: BMC Med Genomics. 2020 Jul 7;13:97. doi: 10.1186/s12920-020-00752-7 (PMC7341625; doi:10.1186/s12920-020-00752-7)

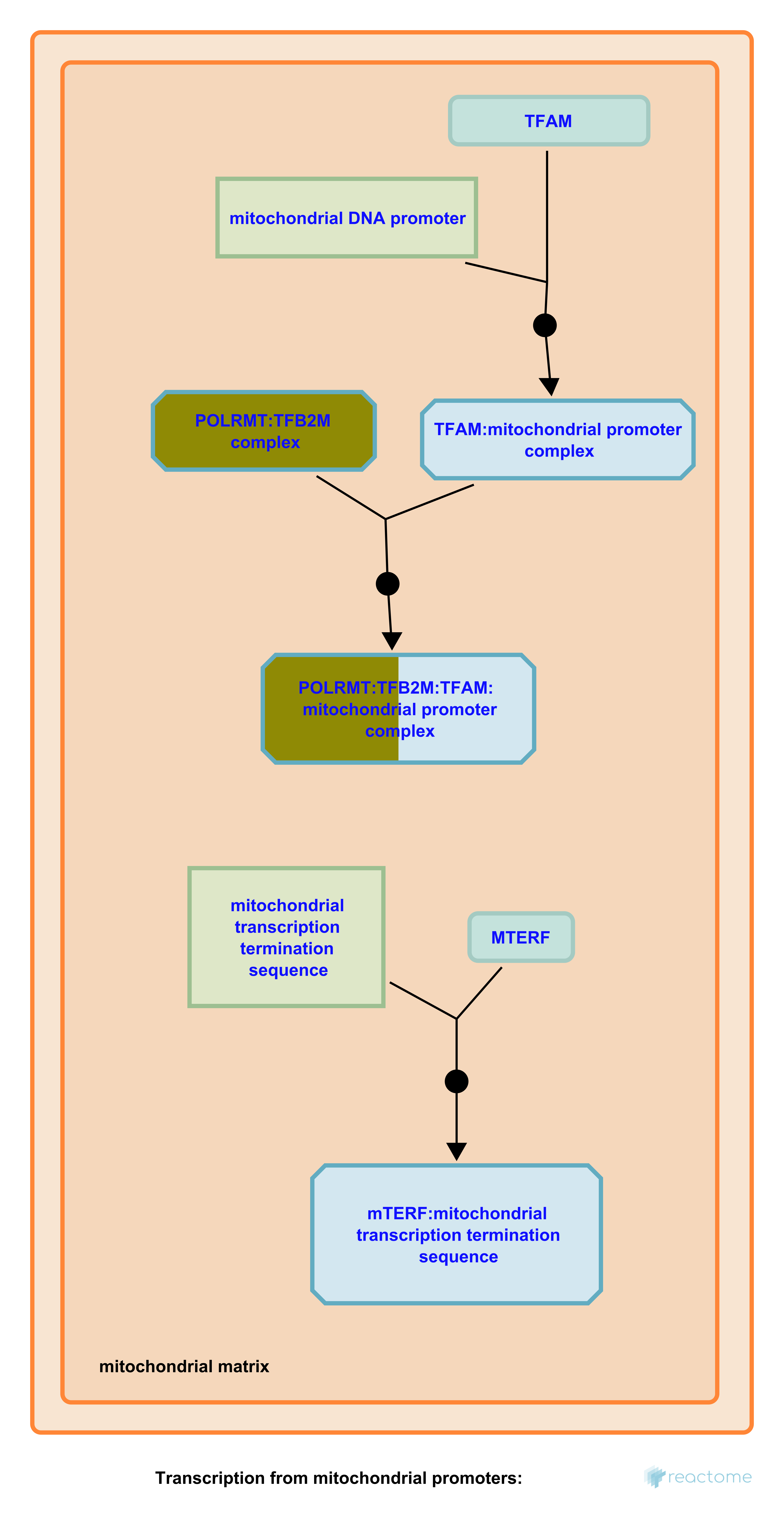

Supplement: Supplementary file 6 — Additional file 6: Figure S1. MitoGWAS on BMI type 1 diabetes patients and additive interactions between mitochondrial and nuclear variants in T1DM patients and FHS cohort. [file 12920_2020_752_MOESM6_ESM.png]
